# Research on Fine‐Grained Phenotypic Analysis of Temporal Root Systems – Improved YoloV8seg Applied for Fine‐Grained Analysis of In Situ Root Temporal Phenotypes

**DOI:** 10.1002/advs.202408144

**Published:** 2024-12-12

**Authors:** Qiushi Yu, Meng Zhang, Liuli Wang, Xingyun Liu, Lingxiao Zhu, Liantao Liu, Nan Wang

**Affiliations:** ^1^ State Key Laboratory of North China Crop Improvement and Regulation College of Mechanical and Electrical Engineering Hebei Agricultural University Baoding 071000 China; ^2^ State Key Laboratory of North China Crop Improvement and Regulation College of Agronomy Hebei Agricultural University Baoding 071000 China

**Keywords:** in situ root system architecture, instance segmentation, phenotype analysis, root image analysis

## Abstract

Root systems are crucial organs for crops to absorb water and nutrients. Conducting phenotypic analysis on roots is of great importance. To date, methods for root system phenotypic analysis have predominantly focused on semantic segmentation, integrating phenotypic extraction software to achieve comprehensive root phenotype analysis. This study demonstrates the feasibility of instance segmentation tasks on in situ root system images. An improved YoloV8n‐seg network tailored for detecting elongated roots is proposed, which outperforms the original YoloV8seg in all network performance metrics. Additionally, the post‐processing method introduced reduces root identification errors, ensuring a one‐to‐one correspondence between each root system and its detection box. The experiment yields phenotypic parameters for fine‐grained roots, such as fine‐grained root length, diameter, and curvature. Compared to traditional parameters like total root length and average root diameter, these detailed phenotypic analyses enable more precise phenotyping and facilitate accurate artificial intervention during crop cultivation.

## Graphical abstract

First, the Improved YoloV8seg network is utilized for fine‐grained segmentation of the collected time‐series root systems. Post‐processing based on Target selection based on the largest connected domain is applied to obtain single‐root masks. In combination with phenotype analysis software, fine‐grained phenotypes of the temporal root systems can be extracted.

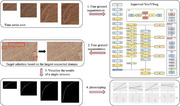

## 1 Introduction

In‐situ root observation methods aim to monitor the growth and development of crop root systems using non‐destructive sampling techniques. These methods include in situ cultivation and in situ imaging techniques. In situ cultivation methods vary based on the cultivation medium, including hydroponics,^[^ ^1^ ^]^ soil culture, gel culture,^[^ ^2^ ^]^ and paper culture.^[^ ^3^ ^]^ Hydroponics, gel culture, and paper culture facilitate direct observation of crop root systems. However, the differences in cultivation media can lead to root morphologies that differ from those grown naturally, and these methods may hinder the formation of root hairs. Gel culture and paper culture are limited by the support capacity of the medium and the nutrient solution content, supporting only the growth of seedling morphology and not allowing for the observation of root systems throughout the full crop life cycle. Soil culture perimits the normal formation of root hairs, yet pose challenges for direct observation of crop roots through the soil.

The most widely used in situ root imaging method is the minirhizotron method.^[^ ^4^ ^]^ The minirhizotron involves inserting a small transparent tube into the soil and obtaining images of crop root systems growing along the tube walls through direct visual observation or camera capture. This method is low‐cost and allows for the automated collection of a large number of images. At the same time, the method of combining minirhizotron images with deep learning to achieve root segmentation has been widely used. For example, Huang et al.^[^ ^5^ ^]^improved OCRNET by GAM to achieve minirhizotron image segmentation, achieving an accuracy of 0.9866, a recall rate of 0.9419, and a precision of 0.8887. Bauer et al.^[^ ^6^ ^]^ used Root Painter and RhizoVision Explorer software to automatically extract features from 58 000 minirhizotron images, and the processing time was 98.1–99.6% faster than manual processing. However, the minirhizotron method also has certain limitations. For example, the installation of the minirhizotron requires the displacement of the soil so that the roots can grow along the tube wall. The root images obtained are somewhat different from the naturally grown root images. At the same time, although the minirhizotron is low‐cost, the clarity of the collected images is also poor. For example, the PRMI minirhizotron dataset^[^ ^7^ ^]^ shows that the root system has low resolution and is blurred. Finally, the minirhizotron cannot observe the structure of the complete root system, which makes it difficult to analyze the root phenotype.

Digital device imaging combines digital devices with growth apparatuses to dynamically collect high‐resolution in situ root images without altering the soil environment or affecting root growth. This method enhances the efficiency of root system segmentation and quantitative analysis.

In the preliminary experiments of this study, precise segmentation of root systems was achieved through an improved UNet,^[^ ^8^ ^]^ and root aging identification was realized by enhancing the Transformer and integrating it with time‐series root images.^[^ ^9^ ^]^

RootNav2.0^[^ ^10^ ^]^ adopts an encoder‐decoder‐based CNN architecture, replacing the previous manual and semi‐automatic feature extraction system RootNav^[^ ^11^ ^]^ with a multi‐task convolutional neural network architecture. It accurately extracts root structures without user interaction and improves speed by nearly 10 times, but requires the root systems to be fully visible for identification. The FaRIA platform^[^ ^12^ ^]^ utilizes an improved UNet structure to segment large‐resolution images into 256 × 256 tiles for prediction, enabling batch prediction of root system images. The RootPainter platform^[^ ^13^ ^]^ includes both semi‐automatic and fully automatic modes. In the semi‐automatic mode, users can subjectively correct each segmented image, and the model can learn from the assigned corrections, reducing segmentation time as the process progresses; the fully automatic mode is more suitable for processing large datasets.

**Figure 1:**
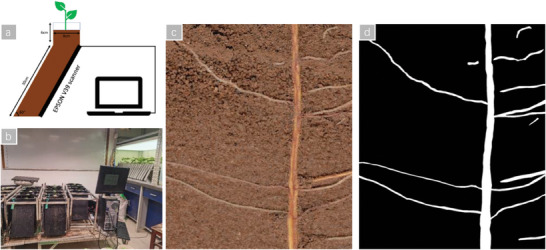
The custom‐made RhizoPot device a) RhizoPot Schematic, b) RhizoPot Device, c) In situ root system images collected, and d) Annotation.

In this experiment, a custom‐made RhizoPot will be utilized. Leveraging the principle of downward root growth, an inclined device is configured. Over time, crop roots will grow closely against an acrylic panel, emerging from the soil. At this point, the roots can be collected using a scanner. This method allows for continuous, non‐destructive collection of comprehensive, high‐resolution in situ root images. **Figure** **1** shows the custom‐made RhizoPot device.

With the development of deep learning techniques, methods for root segmentation based on semantic segmentation networks have become increasingly popular, as these methods more effectively uncover the deep features of targets. SegRoot,^[^ ^14^ ^]^ based on SegNet,^[^ ^15^ ^]^ can distinguish dark soil from roots, though it may exhibit under‐fitting in some cases. Soybean Nodule Acquisition Pipeline (SNAP),^[^ ^16^ ^]^ based on UNet^[^ ^17^ ^]^ and RetinaNet,^[^ ^18^ ^]^ can accurately detect root nodules on soybean roots and segment the primary root. ChronoRoot,^[^ ^19^ ^]^ based on an ensemble model, integrates and averages the results of UNet, ResUNet, DSResUNet, SegNet, and DeeplabV3+,^[^ ^20^ ^]^ achieving a comprehensive architecture and integration of multiple models. In the preliminary experiments of the experimental group, precise segmentation of roots was achieved by improved UNet,^[^ ^8a^ ^]^ and aging recognition of roots was achieved by improving Transformer and combining it with time‐series root images.^[^ ^9^ ^]^

However, there exist shortcomings in root extraction based on semantic segmentation. The results of root segmentation using semantic segmentation are unable to distinguish fine‐grained roots, thereby limiting phenotypic analysis to be understood only from a macroscopic perspective of the entire root system. Presently, most root phenotyping software is based on segmentation results, such as total root length, average root diameter, or maximum surface area. There is a lack of targeted analysis for each lateral root, making it difficult to further interpret the ideal root architecture.

Instance segmentation networks are tasked with detecting and segmenting each target within an image. Prevalent instance segmentation methods include Mask R‐CNN^[^ ^21^ ^]^ and YoloV8seg.^[^ ^22^ ^]^ Mask R‐CNN is a top‐down, two‐stage instance segmentation method. It initially performs detection tasks through the detection head and then segments the detected results through the segmentation head to achieve instance segmentation. YoloV8seg is an improved version based on the one‐stage instance segmentation method YOLACT,^[^ ^23^ ^]^ simultaneously performing detection and segmentation tasks, with results linearly combined to achieve instance segmentation. Both networks represent different implementation methods in the instance segmentation field and can achieve fine‐grained root segmentation tasks.

Tanasaksakul et al.^[^ ^24^ ^]^ tested three instance segmentation algorithms—HTC, Mask R‐CNN, and SOLOV2—on cassava root systems for fine‐grained segmentation. The results showed that deep learning‐based instance segmentation has potential in fine‐grained segmentation of crop roots. However, the cassava images used in this experiment had targets and backgrounds that could be distinguished by the naked eye, without experiments for fine‐grained segmentation of roots in complex environments, necessitating further research. Timely selection of appropriate roots for analysis can help identify the stress conditions of stratified soil in situ roots, allowing for timely artificial intervention to ensure good crop development and further explore the ideal root architecture of crops.

## 2 Experimental Section

### 2.1 Experimental Pipeline

**Figure 2:**
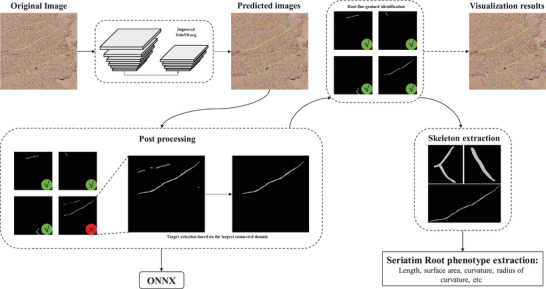
Experimental Procedure.

The experimental procedure of this study is divided into three parts, as shown in **Figure** **2**: (1) Enhancemnt of YoloV8seg for large and elongated targets, including the addition of a large target detection head and the removal of the training target aspect ratio constraint to enable the detection of large, elongated roots. (2) To address the issue of misidentifying other targets during large target prediction, a target selection method based on the maximum connectivity domain was proposed to improve prediction accuracy. (3) For phenotypic analysis of fine‐grained roots, the K3M skeleton extraction algorithm^[^ ^25^ ^]^ was utilized to extract root skeletons and calculate the length, surface area, curvature, and curvature radius of each root, performing curvature analysis on the root systems.

### 2.2 Data Description

#### 2.2.1 Data Collection

This experiment was conducted in 2021 at the experimental station of Hebei Agricultural University in Baoding, Hebei Province, China (38.85°N, 115.30°E). The principles of the image acquisition equipment and the methods of cotton seed cultivation are described in previous articles by the group of this research. Collect topsoil 10–30 cm above the ground and mix it with sandy loam soil in a ratio of 3:1. After air drying and passing through a 20 mesh sieve, it was placed as the culture medium in the RhizoPot device.^[^ ^26^ ^]^ Images of cotton grown in eight RhizoPot equipments were collected using an Epson V39 scanner (Epson Inc., Suwa Shi, Nagano, Japan) starting from the 5th day of growth and subsequently collected every other day for a total of 105 days. The blurred and inconsistent resolution images were removed and then the acquisition of time‐series cotton root system images was realized. The scanner was set to a resolution of 1200 dpi, and the image resolution was 10 200 × 14 039 pixels, saved in Joint Photographic Experts Group format. Continuous images were obtained based on time and group. Twenty in situ root images were selected for annotation, and the remaining 80 images were used for analysis.

#### 2.2.2 Data Annotation

Image annotation was conducted by experienced agronomy experts utilizing the polygon tool in Labelme. The edges of all areas identified as roots were annotated, and once the shapes were closed, they formed the annotated root areas, distinguishing between the primary root and lateral roots. Fine‐grained instances of each category were also annotated. Each image took ≈5 h to annotate, and the results were saved in JavaScript Object Notation (JSON) files. **Table** **1** presents the JSON file objects and descriptions pertinent to this experiment.

**Table 1:** JSON Objects and Descriptions Related to the Experiment.

Object names	Description
shapes	Shape, use “{” and “}” to distinguish shapes formed by different annotation points.
label	Label, indicating the name and nature of the target for training.
points	The coordinates of the target are distinguished by “[” and “]” to distinguish different coordinates.
imagePath	Image path and name.
imageData	The base64 encoding value of the image.
imageHeight	Image height.
imageWidth	Image width.

#### 2.2.3 Data Splitting

Due to the large size of the image datasets, network training is limited by the size of the GPU memory. In this experiment, the in situ root system images were divided into 2048 × 2048 pixels to ensure that the entire root segment could be included as much as possible within the memory constraints. The JSON label files were processed similarly, with the following steps:

Algorithm 1 **Splitting of Json annotated files.**

**Table:** 

**Input**: Json annotation files as *f*;	
Split size as *s*;	
Save path as *savepath*.	
**Input (Optional)**: Root images as *img*.	
**Output**: Split Json files as *f_s_*.	
**1**.	*data* = **json.load**(*f*)
**2**.	*n*1 = 0
**3**.	**for** *i* **in range**(**len**(*data*[′*shapes*′])):
**4**.	*n*2 = 0
**5**.	**for** *j* **in range**(**len(** 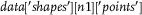 )):
**6**.	*a* = **np.array**($data{[}^{\prime }shapes^{\prime }]\left[n1\right]{[}^{\prime }points^{\prime }]\left[n2\right]$)− **np.array**([*s*, *s*])
**7**.	*data*[′*shapes*′][*n*1][′*points*′][*n*2] = *a* **.tolist**()
**8**.	$x=data{[}^{\prime }shapes^{\prime }\left[n1\right]{[}^{\prime }points^{\prime }\left[n2\right]\left[0\right]$
**9**.	y $=data{[}^{\prime }shapes^{\prime }\left[n1\right]{[}^{\prime }points^{\prime }\left[n2\right]\left[1\right]$
**10**.	**if** *x* > *s* **or** *x* < 0 **or** *y* > *s* **or** *y* < 0:
**11**.	**del** $data{[}^{\prime }shapes^{\prime }\left[n1\right]{[}^{\prime }points^{\prime }\left[n2\right]$
**12**.	*n*2 = *n*2 − 1
**13**.	*n*2 = *n*2 + 1
**14**.	**if** $len\left(data{[}^{\prime }shapes^{\prime }\right]\left[n1\right]{[}^{\prime }points^{\prime }\leq 2$:
**15**.	**del** *data*[′*shapes*′][*n*1]
**16**.	*n*1 = *n*1 − 1
**17**.	*n*1 = *n*1 + 1
**18**.	####**The following are optional actions**####
**19**.	*img*_64_ = **encodeImageForJson**(*img*)
**20**.	*data*[′*imageData*′] = *img*_64_
**21**.	################################
**22**.	$data{[}^{\prime }imageHeight^{\prime }]=s$
**23**.	$data{[}^{\prime }imageWidth^{\prime }]=s$
**24**.	*f_s_* = **json.dump**(*data*, *savepath*)
**25**.	**return** *f_s_*

After the execution of **Algorithm** **1**, the split JSON files will be saved in the savepath, with filenames that correspond to the split images. When optional actions are not used, the value of imageData will be null, thereby conserving storage space and enhancing dataset conversion speed. When optional actions are employed, the value of imageData will contain the corresponding base64 encoded image, enabling the original image and annotations to be visualized in Labelme.

### 2.3 Network Structure and Training Strategy

**Figure 3:**
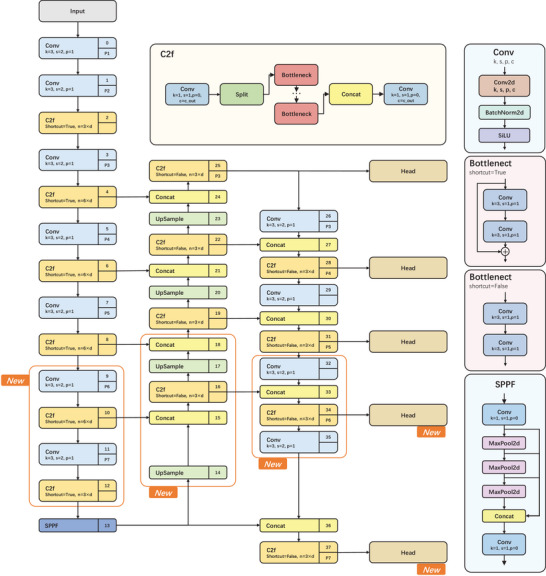
Improved YoloV8seg Network Structure.

To distinguish each segment of the root system in the images, this experiment employed the instance segmentation network YoloV8seg as the root system recognition network. Occasionally, the root systems in the images occupied a substantial portion of the image area, rendering the original YoloV8seg unable to fully recognize these root segments. Consequently, a large target detection method combining multi‐scale detection heads was proposed. First, two additional downsampling layers were added in the backbone part of the network to broaden the receptive field, ensuring thorough extraction of deep features from the images. Then, corresponding to the additional downsampling, two large target detection heads were incorporated to guarantee that the prior boxes completely cover the targets. Finally, considering the elongated structure of the roots, the training strategy was revised by removing the aspect ratio constraint on the targets, enabling a richer more dataset to be utilized for network training. The improved YoloV8seg structure is shown in **Figure** **3**.

### 2.4 Post‐Processing Algorithm

In the prediction results of the enhanced YoloV8seg model, there may occur misidentification of other targets when predicting large targets are being preditced. Some root systems surrounding the long root systems are recognized incorrectly. To address this issue, a target selection method based on the largest connectivity domain has been proposed. The operation process is shown in **Algorithm** **2**.

Algorithm 2 **Target selection based on the largest connected domain.**

**Table:** 

**Input**: Original Mask as *Mask*.	
**Output**: Masks selected through the largest connected domain as *NewMask*.	
**1**.	Perform statistics on the contours in the *Mask* and save the results in a list in *NewMask*.
**2**.	*num* = 0 # Initialize the number of pixels
**3**.	**for** *i* **in** *NewMask*:
**4**.	**if** *len*(*i*) > *num*:
**5**.	*num* = *len*(*i*)
**6**.	**else**:
**7**.	*NewMask*.**delete**(i)
**8**.	**if** *len* (*NewMask*) = = 1:
**9**.	**break**
**10**.	**return** *NewMask*

Following the execution of Algorithm 2, each mask generated by YoloV8seg will exclusively encompass the largest connectivity domain, thereby solving the issue of masks inadvertently encompassing other targets and enabling the extraction of fine‐grained root systems from in situ root images.

### 2.5 Phenotypic Analysis of Fine‐Grained Root Systems

To address the previously unreported challenge of extracting phenotypic parameters for fine‐grained root systems, the outputs of YoloV8seg were processed to ensure that the masks corresponding to all objects in each in situ root system image were saved separately. In this way, each mask image has only one unique object. Next, the RhizoVision Explorer software^[^ ^27^ ^]^was utilized to extract parameters such as root length, surface area, and diameter. The resulting phenotypic parameters represent the root length, surface area, and diameter of each fine‐grained root.

For parameters that the software is unable to directly measure, such as root curvature and curvature radius, the masks of fine‐grained roots underwent a skeletonization process. The K3M skeleton extraction algorithm^[^ ^25^ ^]^ was emplyed to extract the root skeletons, followed by noise reduction. Subsequently, the relevant vector sets were obtained. These vector sets were fitted to obtain the polynomial of the curve. Utilizing the curvature formula, the root curvature and curvature radius were calculated.

(1) $$y=ax^{2}+bx+c\;$$

(2) $$K=\frac{\left|y^{\prime \prime }\right|}{{\left(1+y^{\prime }\right)}^{\frac{3}{2}}}$$

(3) $$\rho =\frac{1}{K}\;\;$$

Equation (1) represents the fitted polynomial, where a, b and c are polynomial coefficients. Equation (2) is the curvature calculation formula, where K is the curvature, y′ is the first derivative of the polynomial, and y″ is the second derivative of the polynomial. Equation (3) represents the formula for calculating the radius of curvature, where ρ is the radius of curvature.

## 3 Results and Discussion

### 3.1 Network Performance Comparison

The study compared the performance of the improved YoloV8n‐seg with the original YoloV8n‐seg, YoloV8s‐seg, YoloV8m‐seg, YoloV8l‐seg, and YoloV8x‐seg. **Table** **2** shows the network performance comparison after 100 epochs of training using an NVIDIA A100 80G GPU, 256G RAM, and an Intel(R) Xeon(R) Gold 6348 CPU, with the batch size set to 1.

**Table 2:** Comparison of network performance metrics (B: Box; M: Mask).

Network	Precision [B]	Recall [B]	mAP50 [B]	mAP50‐95 [B]	Precision [M]	Recall [M]	mAP50 (M)	mAP50‐95 [M]
YoloV8n‐seg	0.69023	0.58895	0.65605	0.41025	0.65669	0.64739	0.66394	0.30567
YoloV8s‐seg	0.69975	0.60347	0.65082	0.40259	0.6911	0.62628	0.63664	0.29719
YoloV8m‐seg	0.77644	0.63983	0.71428	0.45238	0.75551	0.66638	0.71967	0.34578
YoloV8l‐seg	0.80532	0.61681	0.70949	0.45827	0.7822	0.63137	0.71335	0.3485
YoloV8x‐seg	0.77955	0.63363	0.69717	0.44477	0.74505	**0.68057**	0.70925	0.33991
Ours	**0.82194**	**0.67953**	**0.79635**	**0.51176**	**0.8258**	0.64499	**0.74718**	**0.38815**

**Figure 4:**
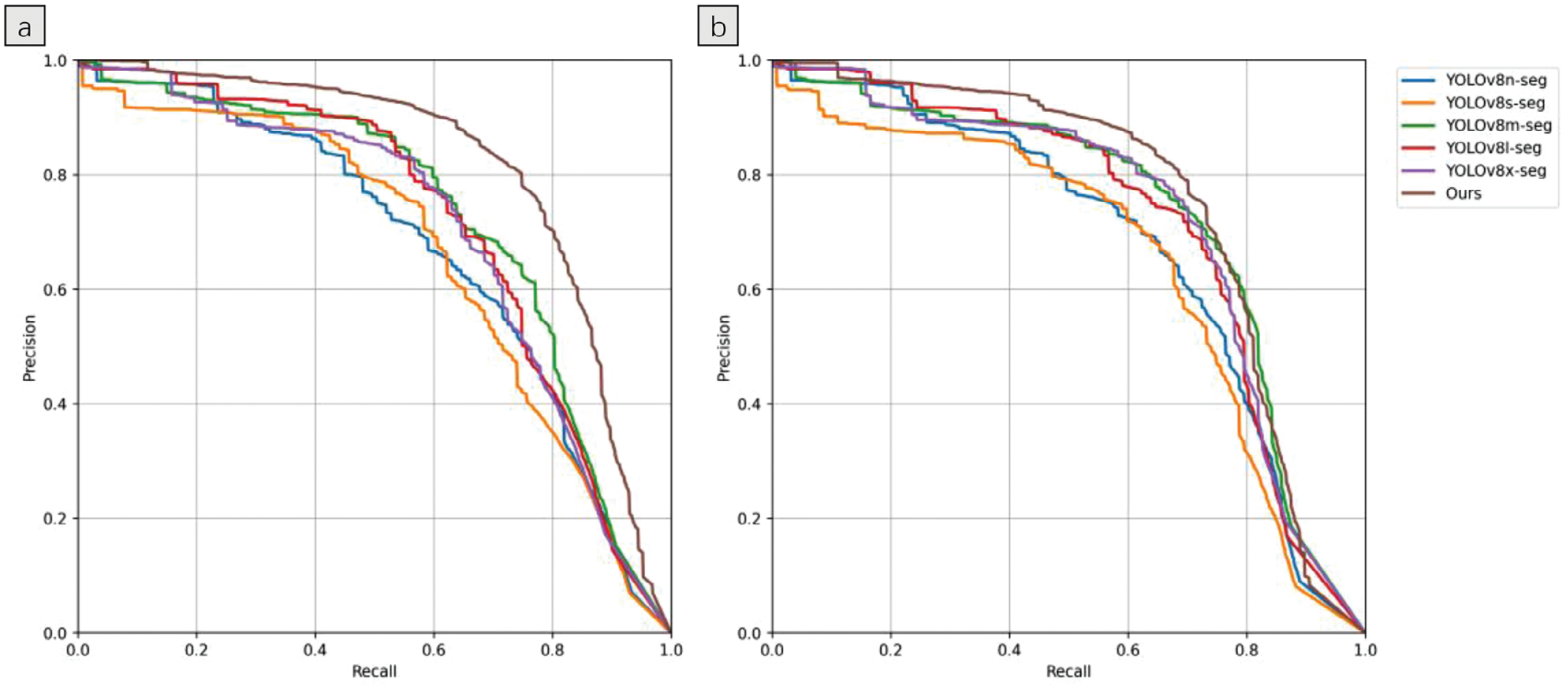
P‐R curve a) P‐R curve for detection task. b) P‐R curve for segmentation task.

From the above table, it can be seen that the improved YoloV8n‐seg reached the highest level in all metrics of detection task and all metrics of recall in segmentation task, only in recall(M) of 0.64499 is lower than the n, m, and x samples of YoloV8seg. To further demonstrate the accuracy of the improved method in this experiment, a Precision‐Recall (P‐R) curve was plotted to describe the predictive performance of the network. **Figure** **4** shows the P‐R curves for various networks in detection and segmentation tasks.

Figure 4a presents the P‐R curve for the detection task. It can be seen that in the detection task, the P‐R curve of the method proposed in this study fully encompasses those of the other networks, suggesting superior performance. Figure 4b displays the P‐R curve for the segmentation task. Although the curve of the method in this study does not fully envelop all the other networks, its settling point, i.e., the point on the line y = x, is closer to the coordinate (1, 1) than the other networks, indicating optimal performance the best in both detection and segmentation tasks.

**Figure 5:**
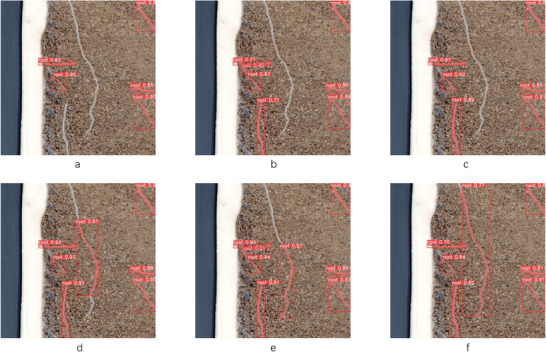
Prediction results of various networks a) YoloV8n‐seg. b) YoloV8s‐seg. c) YoloV8m‐seg. d) YoloV8l‐seg. e) YoloV8x‐seg. f) Ours.

**Figure** **5** illustrates the prediction outcomes of various networks on the test set with a confidence threshold of 0.4. Notably, the improved YoloV8n‐seg effectively predicts longer lateral roots, in contrast to the original YoloV8seg models (n, s, m, l, x) fail to predict the longer lateral roots.

**Table** **3** presents a comparison of the network parameter sizes of the improved YoloV8n‐seg with the original YoloV8seg variants (n, s, m, l, x). Notably, the inclusion of two additional downsampling layers and large object detection heads has resulted in a significantly increase in the number of layers. Despite this, the improved YoloV8n‐seg exhibits lower parameters, gradients, and GFLOPs compared to the other variants, with the exception of the n variant. Similarly, the weight size of the improved YoloV8n‐seg is smaller than those of the s, m, l, and x variants, being only 13.79 MB larger than the n variant.

**Table 3:** Parameters of each network.

Network	Layers	Parameters [M]	Gradients [M]	FLOPs [G]	Size [MB]
YoloV8n‐seg	261	3.26	3.26	12.1	6.91
YoloV8s‐seg	261	11.79	11.79	42.7	23.2
YoloV8m‐seg	331	27.24	27.24	110.4	52.7
YoloV8l‐seg	401	45.94	45.94	220.8	88.4
YoloV8x‐seg	401	71.75	71.75	344.5	137
Ours	431	10.45	10.45	13.8	20.7

In summary, the improved YoloV8n seg outperforms other networks in terms of network performance and prediction accuracy, with only 10.45 parameters slightly lower than YoloV8s‐seg, and 13.8G FLOPs slightly higher than YoloV8n‐seg, making it more convenient to implement ONNX deployment.

### 3.2 Post‐Processing Results

**Figure 6:**
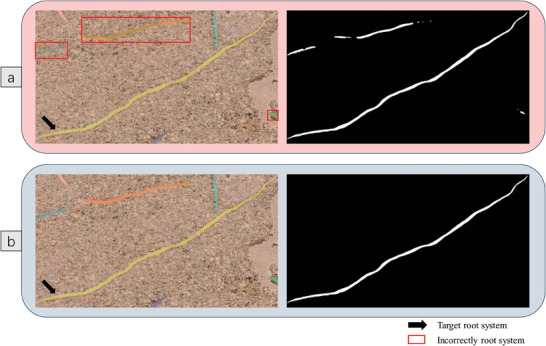
Post‐processing Results a) Prediction results without post‐processing. b) Prediction results with post‐processing.

In the prediction results of the instance segmentation network, there are instances where a long root system may have its detection box encompass part or all of other root systems, resulting in the segmentation head might segment out all the root systems without a clear target, as illustrated in **Figure** **6a**. After the post‐processing steps described in **Section** **2.4**, a detection box will only contain the mask of a single target, as shown in Figure 6b, thereby enabling fine‐grained analysis of each root system.

### 3.3 ONNX Deployment

**Figure 7:**
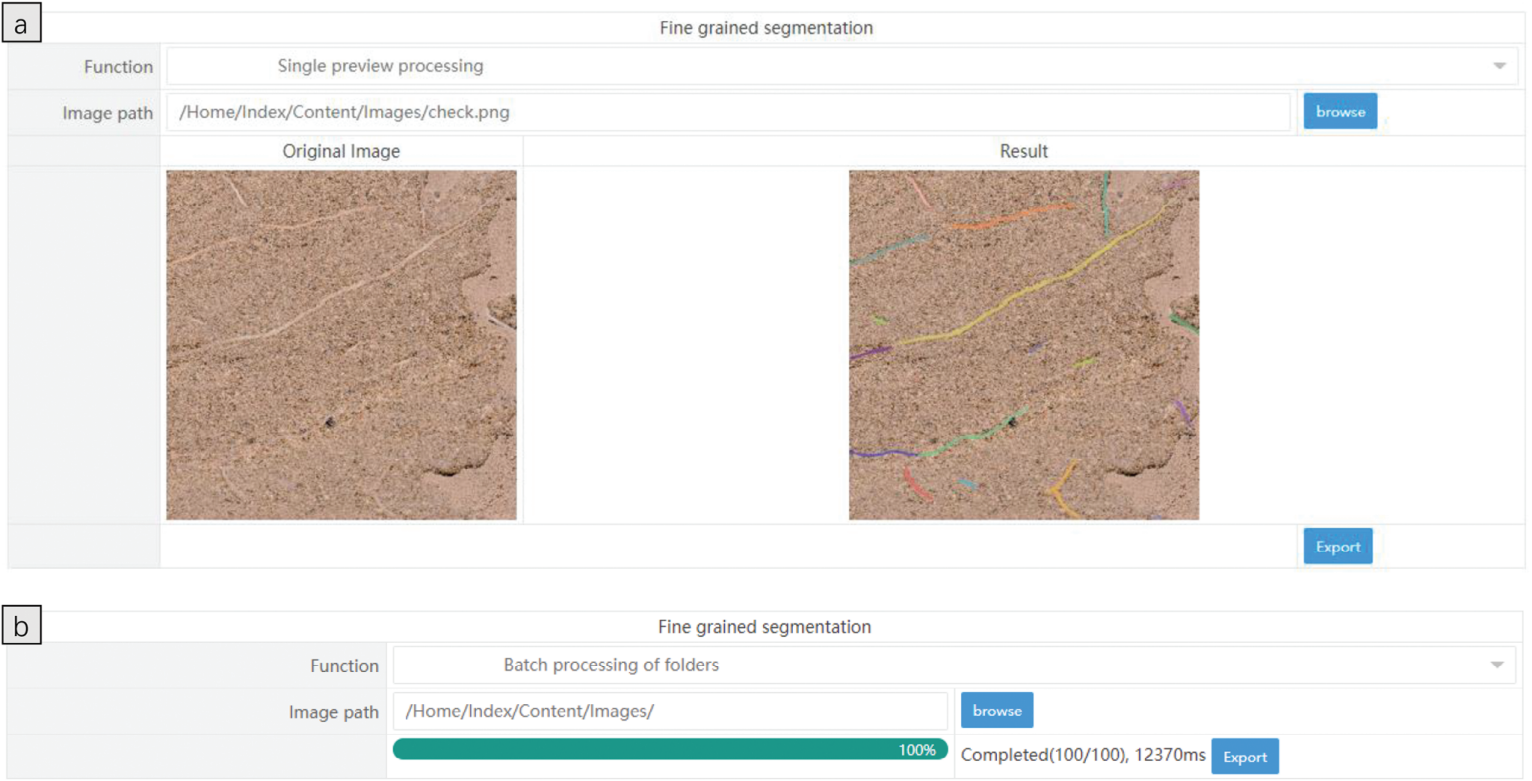
ONNX web interface illustration a) Single preview processing. b) Batch processing of folders.

To broaden the application of the method presented in this study to additional fields, such as the study of field crop root systems, the ONNX exportation of the model output from the experiment was conducted and a user‐friendly visualization interface was designed to enhance human‐computer interaction.  **Figure** **7** illustrates the ONNX visualization interface, where inputting the image path allows for single or batch fine‐grained root segmentation.

Figure 7a demonstrates fine‐grained root system segmentation for a single image. Enter the image path and click the “Predict” button to display the visualization result on the right. The “Instance” shows the number of root system instances detected. Click the “Export” button to export the results locally. Figure 7b shows the fine‐grained segmentation for multiple images. Enter the folder path, wait for the loading to complete, and click the “Predict” button to perform batch segmentation. Upon completion of the batch segmentation, clicking the “Export” button facilitates the local export of the results in a compressed file format.

### 3.4 Phenotypic Parameter Validation

**Figure 8:**
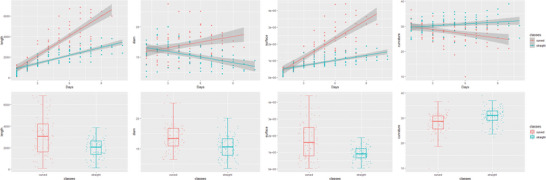
Phenotypic Parameters Analysis: A) Length, B) Diameter, C) Surface Area, and D) Curvature.

Commonly used root system phenotypic parameters encompass root length, max/min/average diameter, and surface area, among others. In this study, it is proposed that root system curvature can also function as a phenotypic parameter indicative of the growth and changes within the root system. Initially, calculations and analyses were conducted on root length, average root diameter, and surface area, with these phenotypic parameters being validated through images depicting growth and development. Extraction of root length, average root diameter, and surface area was facilitated by RhizoVision Explorer software. Upon macroscopic observation, when no further changes in root length were discernible, the root system was deemed to have reached maturity. The timeframe from this point to the emergence of the root system constituted the growth interval, during which the curvature of the root system was computed to monitor changes in root curvature. Through observing the growth state of in situ root system, this study identified two distinct growth patterns: a relatively straight growth pattern and a curved growth pattern. **Figure** **8** displays the results derived from calculations and analyses using the ggplot2^[^ ^28^ ^]^ library in R 4.4.1, chronicling the variations in length, diameter, surface area, and curvature for both growth patterns of the root system. The scatter plot and boxplot depicting curvature represent the outcomes after applying the negative logarithm transformation.

After analysis, this study found significant differences in growth length, diameter, surface area, and curvature between the two states of the root system. For roots with a curved growth pattern, their length, diameter, and surface area increased rapidly. Due to the negative logarithm transformation applied in Figure 8, the curvature exhibited an upward trend. This was because the root systems continued to grow downward, leading to curvature as they bent, resulting in a continuous increase in curvature. Roots with a relatively straight growth pattern showed slower growth in length and surface area. The average root diameter exhibited an overall downward trend due to the growth process. Changes in curvature were slow, as roots with a straight growth pattern were mostly concentrated at the bottom of the RhizoPot device. Initially, the penetration of water was slow for roots with a relatively straight growth pattern. Later, shaded and competed by curved roots, their growth stagnated due to nutrient competition, having already been hindered early on by water scarcity. Additionally, due to nutrient competition in the later stages, their growth stagnated. Consequently, increases in length, surface area, and other metrics were limited, and curvature changes were minimal. The boxplots revealed significant differences in the phenotypic distribution between the two growth patterns. Roots with a curved growth pattern had a more dispersed distribution in length and surface area, indicating faster growth rates. In contrast, roots with a straight growth pattern had a more concentrated distribution in length and surface area, indicating slower growth rates.

### 3.5 Experimental Basis

In the preliminary phase of this experiment, an object detection network combined with a semantic segmentation network was tested for fine‐grained root system segmentation. However, because the BOX of the object detection network encompassed both root systems and soil, and the soil area was significantly larger than the roots, the object detection network was unable to perform fine‐grained detection of the root systems. Therefore, an instance segmentation network was employed for fine‐grained root system segmentation.

The reason for excluding two‐stage top‐down instance segmentation networks in this experiment is that such networks use semantic segmentation for pixel‐level semantic segmentation and then distinguish different instances through clustering, metric learning, etc. This approach is heavily contingent on the quality of the semantic segmentation, necessitates cumbersome post‐processing, and exhibits limited generalization ability. Furthermore, practical applications of this method are confined, leading to the exclusion of top‐down instance segmentation in the present experiment.

**Figure 9:**
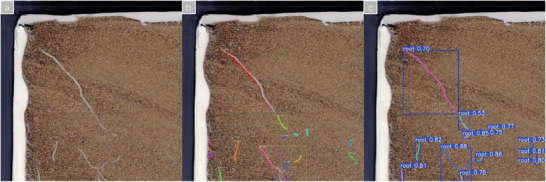
Comparison of the results of the two networks a) Original Image, b) Mask RCNN, and c) YoloV8seg.

Among instance segmentation networks, the Mask R‐CNN network, and the YoloV8seg network were tested. **Figure** **9** shows the comparison of the two network results. The Mask RCNN network may make incomplete predictions when predicting slender roots, and there is an offset in the relative position from the original image. This issue may stem from the top‐down instance segmentation network performing detection first and then segmentation, causing changes in image size when inputting to the segmentation head, thus affecting the segmentation results. In contrast, the YoloV8seg network performs detection and segmentation simultaneously, maintaining the image size unchanged, and its prediction results are closer to the Ground Truth. Therefore, YoloV8seg was selected as the baseline network for improvements.

The YoloV8seg's prediction results were unable to fully enclose large targets within the prediction boxes. Therefore, the improvement plan described in Section 2.3 was proposed. The two additional downsampling layers were added to expand the network's receptive field, and the two large target detection heads were used to detect longer lateral roots. Regarding the training scheme, the original YoloV8seg's training plan included only those labels whose corresponding target boxes had a width and height greater than 0 and an aspect ratio less than 100. The revised training scheme removes the aspect ratio restriction, allowing more elongated roots to be included in the training.

As shown in Figure 5, the improved prediction results are capable of enclosing longer root systems within the detection boxes. The P‐R curves for the detection and segmentation tasks in Figure 4 demonstrate the feasibility of this improvement strategy.

In the preliminary phase of this experiment, the results of adding only one downsampling layer and one detection head was tested. Compared to the original YoloV8seg, the prediction boxes expanded somewhat but did not achieve the goal of fully enclosing the long roots. This may be attributed to the receptive field of network not fully matching the input image size. Therefore, in this experiment, two downsampling layers were utilized in conjunction with two large target detection heads, ensuring the detection boxes fully enclose the roots.

### 3.6 Basis for Phenotypic Extraction

Due to the lack of prior research on root curvature phenotypes, existing phenotypic conclusions were utilized in this study to calculate root curvature, derive its variation patterns, and verify them through data. For curvature extraction, a skeletonization algorithm was employed a skeletonization algorithm was employed to simplify the root systems into their skeletal forms, followed by the calculation of curvature and radius of curvature. The reason for this is that the edge curvature of some roots varies greatly, and direct calculation would yield two different curvatures.  To facilitate statistical analysis, the root skeletonization method was used to obtain a unique curvature representation.

The experiment revealed the phenotypic changes of two main root growth forms in the in situ root image of RhizoPot: the curved growth form of the root system, with more dispersed length and surface area, indicating faster root growth rate. Its curvature shows an upward trend, indicating that the root system is in a downward growth state. And the root system with a relatively straight growth morphology has a concentrated length and surface area, indicating a slower growth rate and a smaller degree of curvature change. This suggests that the root system may have grown relatively straight due to slow water infiltration in the early stage, and later due to obstruction and competition from curved roots.

### 3.7 Prospects

In this experiment, the feasibility of instance segmentation for fine‐grained differentiation of root systems was explored and YoloV8seg was improved to achieve better performance in large object detection. An ONNX deployment was established to make root phenotypic analysis more user‐friendly. Based on previous phenotypic conclusions, the curvature and radius of curvature of root systems were analyzed. However, several limitations of this study should be noted:

1. The root system dataset is singular and not applicable to various crops and planting methods. Future work will involve applying transfer learning methods to extend the results to other datasets and crops. 2. The conclusions about root curvature and radius of curvature obtained in this experiment are only applicable to the analysis of cotton root systems under laboratory conditions. Further analysis is needed for root systems outside the laboratory and under stress conditions. 3. In the early stages of the experiment, the root systems grew too quickly, and some root systems lacked complete growth sequence images. Future studies will utilize root boxes combined with industrial cameras and time‐lapse photography to obtain in situ root images. 4. Fine‐grained root system differentiation based on instance segmentation networks has inherent advantages over semantic segmentation‐based root segmentation.  Future work will involve analyzing root system aging and different soil depths. 5. The current in situ root fine‐grained segmentation webpage has limited functionality. Future enhancements will include adding location information of in situ root system images, displaying the position of root images on the original image to observe root growth trends more intuitively. 6. The root structure of cotton cultivated based on RhizoPot is different from that in the field, and most of the roots in the device are in a flat plane, lacking a 3D configuration. Due to the limited capacity of the device, the root system growing inside RhizoPot is shorter than that in the field. Future improvements to the root collection device will aim to make the root growth structure more representative of field roots.

Further exploration of the method proposed in this article will help to clarify the growth morphology of in situ roots. At the same time, fine‐grained extraction of roots will aid in analyzing the phenotypic parameters of each root system, helping to determine whether the entire crop or roots at different depths are under stress. This will enable more precise artificial intervention and achieve optimal crop production and development.

## 4 Conclusion

This experiment achieves fine‐grained segmentation of in situ root systems using instance segmentation. Compared to traditional semantic segmentation networks, instance segmentation networks can distinguish each segment of the root system and extract phenotypic data for each segment. This paper proposes an improved YoloV8n‐seg network for detecting elongated root systems, with performance metrics surpassing the original YoloV8seg. Additionally, the proposed post‐processing method reduces root system recognition errors, ensuring that each root system corresponds to a single detection box. The phenotypic parameters obtained from this experiment, such as fine‐grained root length, root diameter, and curvature, enable more precise phenotypic analysis compared to traditional metrics like total root length and average root diameter. This precision facilitates the identification of root system stress in layered soils and supports precise artificial intervention during crop cultivation.

## Conflict of Interest

The authors declare that there are no conflicts of interest.

## Author Contributions

L.L., N.W., and M.Z. initiated and designed the research. Q.Y., M.Z., L.W., X.L., and L.Z. performed the experiments and collected the data. Q.Y. wrote the code and tested the methods. Q.Y. and M.Z. analyzed the data and wrote the manuscript. All authors contributed to the article and approved the submitted version.

## Data Availability

The data that support the findings of this study are available from the corresponding author upon reasonable request.
